# The Dual Antioxidant/Prooxidant Effect of Eugenol and Its Action in Cancer Development and Treatment

**DOI:** 10.3390/nu9121367

**Published:** 2017-12-17

**Authors:** Daniel Pereira Bezerra, Gardenia Carmen Gadelha Militão, Mayara Castro de Morais, Damião Pergentino de Sousa

**Affiliations:** 1 Instituto Gonçalo Moniz, Fundação Oswaldo Cruz (IGM-FIOCRUZ/BA), Salvador 40296-710, Bahia, Brazil; danielpbezerra@gmail.com; 2 Departamento de Fisiologia, Universidade Federal de Pernambuco, Recife 50670-901, Pernambuco, Brazil; gcgadelha@yahoo.com.br; 3 Departamento de Ciências Farmacêuticas, Universidade Federal da Paraíba, João Pessoa 58051-970, Paraíba, Brazil; mayaracastrodemorais@gmail.com

**Keywords:** reactive oxygen species, metabolism, antitumor activity, antioxidant activity, phenylpropanoid, natural products, essential oils, clove, *Syzygium aromaticum*

## Abstract

The formation of reactive oxygen species (ROS) during metabolism is a normal process usually compensated for by the antioxidant defense system of an organism. However, ROS can cause oxidative damage and have been proposed to be the main cause of age-related clinical complications and diseases such as cancer. In recent decades, the relationship between diet and cancer has been more studied, especially with foods containing antioxidant compounds. Eugenol is a natural compound widely found in many aromatic plant species, spices and foods and is used in cosmetics and pharmaceutical products. Eugenol has a dual effect on oxidative stress, which can action as an antioxidant or prooxidant agent. In addition, it has anti-carcinogenic, cytotoxic and antitumor properties. Considering the importance of eugenol in the area of food and human health, in this review, we discuss the role of eugenol on redox status and its potential use in the treatment and prevention of cancer.

## 1. Introduction

Reactive oxygen species (ROS) are a heterogeneous group of molecules that are, along with endogenous antioxidants, ubiquitously present in all organisms. They are implicated in various diseases including malignant transformations [1]. The term “oxidative stress” refers to an imbalance in which pro-oxidants overwhelm the capacity of antioxidant defense systems [2]; it has been shown to contribute to the development of some types of cancer [3]. 

**Figure 1:**
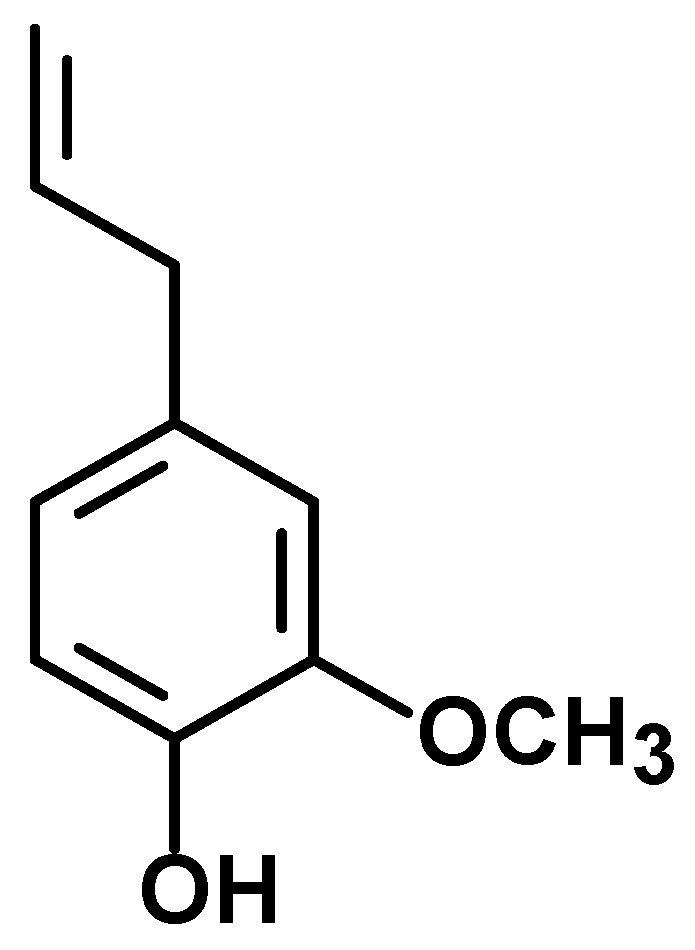
Chemical structure of eugenol.

The report of anticancer potential of aromatic compounds found in foods and plants have increased in the recent decades [4,5,6,7,8] and there are advanced studies of mechanisms of action and clinical approaches in progress. This chemical class of natural products show interesting potential as health promoting agents and, consequently, with application to improving the quality of life. These include the polyphenols that are important components of human diet. Interestingly, some of these compounds may act as either antioxidants or pro-oxidants to exert protective effects against cancer [9,10,11]. Eugenol (4-allyl-2-methoxyphenol) (Figure 1) is an aromatic phenylpropanoid phenol contained in clove (*Syzygium aromaticum*, Myrtaceae), which is well-known for its culinary uses. Eugenol also occurs in soybeans, mung beans [12], coffee [13], bananas [14] and in herbs such as nutmeg (*Myristica fragrans*, Myristicaceae), cinnamon (*Cinnamomum verum*, Lauraceae) and basil (*Ocimum basilicum*, Lamiaceae); however, *Syzygium aromaticum* can be considered the principal natural source of this compound (45% or 90% of the total oil) [15]. Clove has been used for a long time by civilizations because of its flavor and its properties make it important for culinary and medicinal uses. Eugenol has been included as a spicy flavoring in whisky, ice cream, baked goods and candy in restricted concentrations [16,17,18]. Eugenol has dual effect on the oxidative stress, which can action as an antioxidant or prooxidant agent. In addition, it has anti-carcinogenic, cytotoxic and antitumor properties. Considering the importance of eugenol in the area of food and human health, in this review, we discuss the role of eugenol on redox status and its potential use in the treatment and prevention of cancer. Searches were performed in the scientific literature database PubMed comprising all papers in English published until September 2017 using the following key words: eugenol with oxidant; antioxidant; cancer; cytotoxic; or antitumor. No exclusion criteria were performed.

## 2. Anti-Carcinogenic/Chemopreventive Effect of Eugenol and Its Relation to the Inhibition of Oxidative Stress

**Figure 2:**
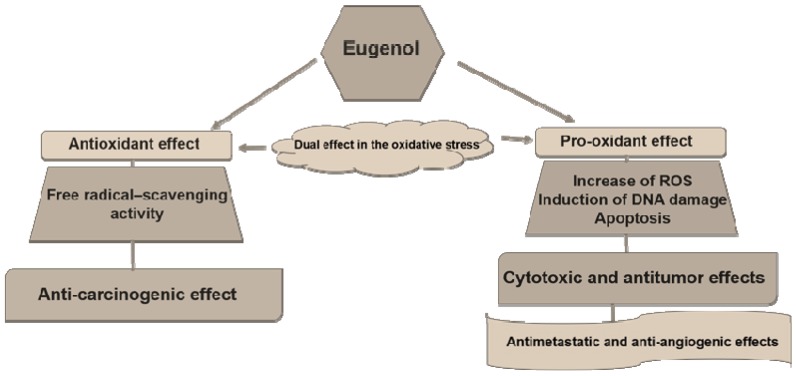
The dual effect of eugenol in the oxidative stress and its action in cancer development and treatment.

The ability to inhibit oxidative stress has been described as a protective effect against cancer formation (carcinogenesis or tumorigenesis); on the other hand, once a cancer has already formed, the antioxidant effect can contribute to the cancer’s development, while the pro-oxidant effect can induce cancer cell death by several signaling pathways [19]. Interestingly, eugenol has been described as an agent with a double effect, antioxidant and pro-oxidant, presenting beneficial effects in the prevention of cancer formation and in cancer treatment (Figure 2). Despite some contradictory studies, there are many articles evaluating these biochemical and pharmacologic aspects.

The anti-carcinogenic effect of eugenol had been investigated in several models [20,21,22,23,24,25,26,27,28]. The anti-carcinogenic effect of eugenol against skin carcinogenesis was investigated by Kaur et al. [20]. Skin cancer was initiated by applying 160 nmol 7,12-dimethylbenz[a]anthracene (DMBA) and promoted by twice weekly applications of 8.5 nmol 12-otetradecanoylphorbol-13-acetate (TPA) for 28 weeks and was followed by eugenol treatment. DMBA is a polycyclic aromatic hydrocarbon pro-carcinogen that requires metabolic conversion to its ultimate carcinogenic diol epoxide metabolites by oxidation, which is carried out through cytochrome P450 family 1 subfamily A member 1 (CYP1A1) and cytochrome P450 family 1 subfamily B member 1 (CYP1B1). Therefore, the carcinogenic effect of DMBA depends on the level of the oxidative metabolism of cytochrome P450 family 1. Two protocols were established: an anti-initiation protocol (topical application of 200 µL eugenol at 15% *v*/*v* in acetone one week before, one hour prior and two times after DMBA application); and an anti-promotion protocol (topical application of 30 µL eugenol at 15% *v*/*v* in acetone, 30 min prior to every TPA application). The treatment with eugenol did not prevent tumor formation but led to a reduction in tumor size. The control group presented tumor size of 9.7 g, and eugenol treatment showed tumor size of 5.6 g in the anti-initiation protocol and 2.8 g in the anti-promotion protocol. In addition, topical application of eugenol prior to TPA exposure led to the development of papillomatous keratoacanthoma with minimal cell proliferation but without squamous cell carcinoma. The anti-carcinogenic effect of eugenol was attributed to its anti-inflammatory activity, because some markers of inflammation, including inducible nitric oxide synthase (iNOS) and cyclooxygenase-2 (COX-2) expression and the levels of pro-inflammatory cytokines interleukin-6 (IL-6), tumor necrosis factor alpha (TNF-α) and prostaglandin E2 (PGE2), were reduced in DMBA/TPA-exposed animals after treatment with eugenol. Furthermore, eugenol was found to suppress the activation of nuclear factor kappa B (NF-κB) in mouse skin with TPA-induced inflammation [20].

Additionally, eugenol treatment (~100 mg/kg) inhibited the tumor formation in mouse skin model induced by application of DMBA as initiator and croton oil as promotor via radical scavenging activity of eugenol, downregulation of Myc (proto-oncogene), H-ras (harvey rat sarcoma virus oncogene) and Bcl-2 (B-cell lymphoma 2, apoptosis regulator) expression along with upregulation of p53, Bax (BCL2 associated X, apoptosis regulator) and active caspase-3 expression in the skin lesions [21,22]. Topical administration of eugenol also partially inhibited the benzo[a]pyrene-induced skin carcinogenesis in Swiss mice [23]. However, topical application of eugenol had minimal protection in reducing DMBA-induced skin carcinogenesis in Swiss mice [24].

The chemopreventive effect of eugenol on *N*-methyl-*N*′-nitro-*N*-nitrosoguanidine (MNNG)-induced gastric carcinogenesis in Wistar rats was also performed [25,26]. MNNG (150 mg/kg) was administered by intragastric intubation three times with a gap of two weeks in between the treatments and eugenol (100 mg/kg) was administered by intragastric route, three times per week starting on the day following the first exposure to MNNG and continued until the end of the experimental period. The incidence of gastric tumors in MNNG-treated rats was 100% with a mean tumor burden of 274.38 mm^3^ and eugenol treatment decreased the tumor incidence to 16.66% with a tumor burden of 14.78 mm^3^. Administration of eugenol induced apoptosis via the mitochondrial pathway by modulating the Bcl-2 family proteins, apoptotic protease activating factor 1 (Apaf-1), cytochrome c and caspases and inhibiting of invasion and angiogenesis as evidenced by changes in the activities of matrix metalloproteinases (MMP) and the expression of MMP-2 and -9, vascular endothelial growth factor (VEGF), vascular endothelial growth factor receptor 1 (VEGFR1), tissue inhibitor of metalloproteinase-2 (TIMP-2) and reversion-inducing-cysteine-rich protein with kazal motifs (RECK). Moreover, reduction in the NF-κB activation along with increasing of its inhibitor family members, IκB kinase α (IκBα) and inhibitor of kappa B (IKKβ), reduction of cyclin D1, cyclin B and proliferating cell nuclear antigen (PCNA) and increasing of p53, p21^waf1^ and growth arrest and DNA damage-inducible 45 (Gadd45) were observed in eugenol-treated animals [25,26].

Using MCF 10A breast epithelial cells and H-ras transfected MCF 10A (MCF 10A-ras) as a model of cancer progression, eugenol exhibited cytotoxicity in µM range to MCF 10A-ras cells but not in MCF 10A cells [27]. In addition, eugenol reduced the ATP generation and inhibited oxidative phosphorylation and fatty acid oxidation via downregulating of c-Myc/PGC-1β/ERRα signaling pathway and inhibiting ROS production in H-ras transfected MCF 10A breast epithelial cells, indicating that eugenol can prevent breast cancer progression by regulation of cellular energy metabolism [27]. On the other hand, eugenol treatment does not exert modifying effects on lung carcinogenesis induced by urethane [28]. No significant differences in the incidences and multiplicities of lung lesions were observed between eugenol and control groups. In this model of lung carcinogenesis, transgenic mice with the human prototype c-Ha-ras gene received a single intraperitoneal injection of 250 mg/kg urethane, followed by a diet containing 6000 ppm eugenol or basal diet for 26 weeks [28]. The Table 1 summarize the anti-carcinogenic effect of eugenol.

The anti-carcinogenic effect of eugenol can also be attributed to its antioxidant property. Eugenol has been reported to have antioxidant activity, as assessed by diverse models [12,29,30,31,32,33,34,35]. Eugenol reacts with 2,2-diphenyl-1-picrylhydrazyl (DPPH) and shows high DPPH free radical-scavenging activity [29,30,31,32,33]. The concentration of eugenol required for 50% DPPH scavenging (IC_50_: half maximal inhibitory concentration) activity ranged from 98 to 138 µM [31,35]. Eugenol also exhibits effective antioxidant activity in the linoleic acid emulsion system by inhibiting lipid peroxidation at 91 µM. In addition, eugenol has ferric ion (Fe^3+^) reducing ability and electron donor properties for neutralizing free radicals by forming stable products [31]. Eugenol inhibits malonaldehyde (MA) formation from cod liver oil by 91% at ~1 mM [12]. Furthermore, eugenol inhibits microsomal lipid peroxidation (IC_50_ about 80 µM) as well as iron and OH radical-initiated lipid peroxidation in rat liver mitochondria, with IC_50_ values of 10 and 14 μM, respectively. The antioxidant effect was determined by the inhibition of thiobarbituric acid-reactive substances (TBARS) formation [29,30].

The effect of eugenol on in vivo lipid peroxidation mediated by carbon tetrachloride (CCl_4_) has also been evaluated [30]. The CCl_4_ model has been used for many years to investigate the effect of antioxidants in the liver xenobiotic metabolism. When eugenol was given at 5 mg/kg orally at three different times—i.e., prior to (−1 h), along with (0 h), or after (+3 h)—in relation to the time of CCl_4_ dosing (i.p. administration of 0.4 mg/kg), it prevented significantly the rise in serum glutamic-oxaloacetic transaminase (SGOT) activity, lipid peroxidation and liver necrosis. However, eugenol failed to prevent a decrease in glucose-6-phosphatase activity, suggesting that the damage to endoplasmic reticulum (ER) is not protected by eugenol. Thus, the protective action of eugenol can be explained by the interception of secondary radicals derived from ER lipids rather than interference with the primary radicals of CCl_4_ (•CCl_3_/CCl_3_OO•) [30]. In addition, the in vivo antioxidant effect of eugenol on liver danger induced by thioacetamide (TA) was also performed [36]. TA is frequently used to produce liver danger in animals due to generation of ROS and instigation of oxidative stress, which causes liver damage. Adult male Wistar rats were treated with eugenol (10.7 mg/kg/day) orally for 15 days. TA was administered (300 mg/kg, i.p.) for the last two days at 24 h intervals and the rats were sacrificed on the 16th day. Pretreatment with eugenol controlled the levels of lipid peroxidation and protein oxidation products with consequent reduction of TBARS, lipid hydroperoxides and protein carbonyl formation in plasma and the liver. Increased expression of the COX-2 gene as well as increases in pro-inflammatory cytokine TNF-α and IL-6 plasma levels induced by TA was also partially reverted by eugenol pretreatment. The protective effect of eugenol can be attributed to the reduction of cytochrome P450 family 2 subfamily E member 1 (CYP2E1) activity, the main enzyme responsible for TA-induced hepatotoxicity and oxidative stress [36].

Genotoxicity and mutagenicity of xenobiotics are also involved in the carcinogenic process and may occur as a result of oxidative stress. Interestingly, the antimutagenic and anti-genotoxic effects of eugenol has been also reported. Eugenol suppressed the mutagenicity induced by furylfuramide, 4-nitroquinoline 1-oxide, aflatoxin B in *Salmonella typhimurium* [37]. Eugenol also inhibits detoxification enzymes and prevents DMBA-induced DNA damage in MCF-7 (human breast adenocarcinoma) cell line [38,39]. Eugenol at dose of 50–500 mg/kg administered by gavage prevents the genotoxicity-induced by cyclophosphamide, procarbazine, *N*-methyl-*N*′-nitro-*N*-nitrosoguanidine and urethane [40]. In addition, the mutagenicity of benzo[a]pyrene but not DMBA and aflatoxin B1, in the *S. typhimurium* mutagenicity assay was reduced in liver S-9 fractions prepared from rats treated orally with eugenol (1000 mg/kg) [41]. In contrast, eugenol causes intrachromosomal recombination in yeast *Saccharomyces cerevisiae* in logarithmic phase cultures [42] and although eugenol induces no mutagenesis in Ames test, it causes chromosomal aberrations and increased the incidence of sister chromatid exchanges in Chinese hamster ovary cells [43,44]. At µM range, eugenol is not able to prevent the DNA lesions induced by hydrogen peroxide (H_2_O_2_) [33]. However, eugenol protected the supercoiled pBR322 plasmid DNA oxidative damage induced by Fe^2+^ and H_2_O_2_ at mM range [35]. Moreover, eugenol, at concentrations above 50 µM, inhibited the DNA oxidative damage induced by hydroxyl radicals produced by Fenton reactions using Fe^2+^ and H_2_O_2_ [32].

## 3. Cytotoxic and Antitumor Effects of Eugenol and Its Relation to the Induction of Oxidative Stress

Controversial results have been found for the cytotoxic activity of eugenol. Some studies have shown that eugenol is capable of inducing cytotoxicity at concentrations in the μM range, whereas other studies show that eugenol is capable of inducing cytotoxic effects only at concentrations in the mM range. Nevertheless, eugenol is able to induce cytotoxicity to cancer cell lines with different histological types, including skin, breast, colon, prostate, cervical, hepatocellular, lung, oral squamous cells and leukemia. In addition, the ability to induce oxidative stress has been also ascribed to eugenol in cell-based assays.

Eugenol in the μM range inhibits the growth of melanoma cells—Sbcl2 (primary melanoma), WM3211 (primary radial growth phase), WM98-1 (primary vertical growth phase) and WM1205Lu (metastatic melanoma)—accompanied by cell cycle arrest at the S phase, followed by apoptosis [45]. Using cDNA array analysis, it was demonstrated that eugenol modulates expression of E2F family members. In addition, eugenol was able to inhibit the E2F1 transcriptional activity and, as overexpression of E2F1 restores melanoma cell proliferation, this indicates that eugenol targets E2F functions in melanoma cells [45]. In addition, eugenol in the μM range inhibits the growth of HL-60 (human promyelocytic leukemia), U-937 (human histiocytic lymphoma), HepG2 (human hepatocellular carcinoma), 3LL (Lewis mouse lung carcinoma) and SNU-C5 (human colon carcinoma) lines [46]. Eugenol-treated HL-60 cells display DNA fragmentation, ROS production, loss of mitochondrial transmembrane potential, bax translocation, Bcl-2 reduction, cytochrome c release and caspase-9 and -3 activation, suggesting that eugenol causes apoptotic cell death. Moreover, pretreatment of HL-60 cells with *N*-acetyl-l-cysteine (an antioxidant), Z-VAD-FMK (a pan caspase inhibitor) and Z-DEVD-FMK (a caspase-3 inhibitor) decreases the eugenol-induced apoptosis, indicating that eugenol activates the caspase- and ROS-mediated apoptosis pathways [46]. Moreover, treatment of HL-60 cells with eugenol, produced formation of three DNA adducts and incubation of HL-60 cells with the combination of 100 μM eugenol and 100 μM H_2_O_2_ potentiated the levels of DNA adduct in HL-60 cells. Oxidative base damage was also observed. The DNA adducts formed were inhibited by the addition of either ascorbic acid or glutathione [47]. Eugenol in the μM range is also cytotoxic to DU-145 (androgen-insensitive prostate cancer cells) and KB (oral squamous carcinoma cells) [48].

Using LNCaP (androgen responsive human prostate carcinoma) and PC-3 (androgen independent human prostate carcinoma) cell lines, eugenol induces cytotoxicity in the μM range and causes an increase in G_2_/M phase [49]. Apoptotic cell death was not detected at the concentrations used; however, eugenol in combination with 2-methoxyestradiol causes apoptosis along with a reduction of the expression of anti-apoptotic protein Bcl-2 and enhancement of the expression of the pro-apoptotic protein Bax. The apoptosis induced by this combination is not affected in PC-3 cells with overexpression or lack of Bcl-2 but is associated with the loss of mitochondrial membrane potential [49]. Eugenol in μM concentrations causes cytotoxicity to MCF-7, T47-D (human breast carcinoma) and MDA-MB-231 (human breast adenocarcinoma) cells through down-regulation of E2F1 and its downstream anti-apoptosis target, surviving independently of the status of p53 and ERα [50]. Eugenol inhibits the breast cancer related oncogenes, NF-κB and cyclin D1 and up-regulates the cyclin-dependent kinase inhibitor p21^WAF1^ protein; On the other hand, eugenol was also cytotoxic to non-cancer cell line MCF 10A (human breast epithelial) with IC_50_ value of 2.2 μM [50]. Júnior et al. [51] also assessed the cytotoxicity of eugenol in the μM range on MDA-MB-231, MCF-7, SIHA (human cervix carcinoma), SK-Mel-28 (human melanoma) and A2058 (human melanoma) cells; it was accompanied by ROS production, causing G2/M phase block and, consequently, clastogenesis. Eugenol also induced downregulation of PCNA (proliferation cell nuclear antigen), decreased the mitochondria transmembrane potential and upregulated Bax [51].

Controversially, some studies have indicated that eugenol has no cytotoxic activity or has cytotoxicity only when present in the mM range [52,53,54,55,56,57,58,59,60,61,62,63,64,65,66,67,68,69,70,71,72,73]. In studies with HSG (human submandibular gland adenocarcinoma) and HSC-2 (human oral squamous cell carcinoma) cells, eugenol caused cytotoxicity when in the mM range but no ROS induction was observed [58,59]. On the other hand, Atsumi et al. [60] stated that eugenol caused a biphasic ROS production that was enhanced at 5–10 μM and decreased at 500 μM in HSG, treated with H_2_O_2_ plus horseradish peroxidase or with visible light irradiation. In HL-60 cells, eugenol presents IC_50_ of 0.38 mM that is accompanied by internucleosomal DNA fragmentation. The expression of the mRNAs and the activity of manganese superoxide dismutase and copper- and zinc-containing superoxide dismutase are inhibited by eugenol, suggesting that eugenol targets the oxidative stress in cancer cells. In contrast, eugenol-induced cytotoxicity is enhanced by *N*-acetyl-l-cysteine or glutathione treatment [58,61]. On the other hand, eugenol induces cytotoxicity and ROS generation in HSG cells in which glutathione or cysteine are protecting from damage [62,63].

Pisano et al. [64] demonstrated that eugenol has no cytotoxic effect at 100 μM in malignant melanoma cell lines WM266-4, SK-Mel-28, LCP-Mel, LCM-Mel, PNP-Mel, CN-MelA, 13443 and GR-Mel. In human melanoma G361 cells, eugenol in the mM range inhibits the viability of G361 cells. Eugenol-treated G361 cells present caspase-3 and -6 cleavage and activation. The caspase-3 substrates poly(ADP-ribose)polymerase (PARP) and DNA fragmentation factor 45 (DFF45) are cleaved in eugenol-induced apoptosis, suggesting induction of caspase-dependent apoptosis [65]. Interestingly, similar results were found in human osteosarcoma HOS cells, suggesting that eugenol can also induce caspase-dependent apoptosis pathways in HOS cells [66].

In a study with human cervical carcinoma cell line (HeLa), eugenol presented cytotoxicity in the mM range and in a synergistic combination with sulforaphane, downregulated the expression of Bcl-2, COX-2 and IL-β. It also produced a synergistic effect when combined with gemcitabine, causing downregulation of the expression of Bcl-2, COX-2 and IL-β [67,68]. Also, in the mM range, eugenol was cytotoxic and induced apoptosis to colon carcinoma cell lines HCT-15 and HT-29. The loss of the membrane mitochondrial potential and generation of ROS were accompanied in the eugenol-induced apoptosis. Augmented ROS generation resulted in the DNA fragmentation and activation of PARP, p53 and caspase-3 [69]. Eugenol in the mM range also inhibits the growth of human breast carcinoma MCF-7 cells, accompanied by cell shrinkage and an increase in the percentage of apoptotic cells and DNA fragments. A depleted level of intracellular glutathione and increased level of lipid peroxidation are also observed [70]. In another study with MCF-7 cells, eugenol presented an IC_50_ value of 0.9 mM, increased the ROS production, decreased the ATP level and induced the loss of the mitochondrial membrane potential and release of the cytochrome c and lactate. Cell viability and ROS production were restored by pretreatment with the antioxidants. On the other hand, the eugenol effect was not affected in MCF-7 cells with overexpression of Bcl-2 [71]. Human oral squamous cell carcinoma cell line HSC-2 treated with a concentration of eugenol in the mM range presented metabolic changes including reduction of ATP utilization, oxidative stress and an increase in the polyamines and glycolytic metabolites [72]. In HepG2 and Caco-2 (human colon carcinoma) cells, the treatment with eugenol-loaded nanoemulsions and free eugenol caused increasing in the cell death by apoptosis and ROS generation [73]. The Table 2 summarize the in vitro cytotoxic effect of eugenol.

In vivo antitumor effects of eugenol have been also investigated [45,50,74]. Using B6D2F1 mice bearing B16 melanoma, eugenol treatment (125 mg/kg/i.p. of body weight twice a week) caused the in vivo antitumor effect [45]. On day 15, the size of tumors in the eugenol-treated group was 62% less than the control group, with an increase of 19% in the survival rate. At the end of the treatment, 50% of the animals in the control group presented metastases but no eugenol-treated animals showed any signs of invasion or metastasis [45]. Moreover, eugenol (100 mg/kg/i.p.) was able to inhibit the growth of the Ehrlich ascites model by 28.88% and inhibited 24.35% tumor growth in the Ehrlich solid tumor model [74].

In mice engrafted with human breast adenocarcinoma MDA-MB-231 cells subcutaneously, eugenol treatment with a dose of 100 mg/kg every two days for four weeks inhibited tumor growth [50]. Moreover, eugenol downregulated E2F1, survivin, NF-κB and cyclin D1 and increased the levels of p21^WAF1^, Bax, cleaved PARP-1 and the active form of caspase-9 in tumor xenografts [50]. The Table 3 summarize the in vivo antitumor effect of eugenol. Regarding the antimetastatic potential of eugenol, it exerts inhibitory effects on matrix metallopeptidase 9 (MMP-9) via inhibition of extracellular signal-regulated kinase (ERK) phosphorylation in human fibrosarcoma HT1080 cells [32].

Although there are a large number of papers on the cytotoxic properties of eugenol, controversial results delay the completion of preclinical efficacy and safety studies as well as clinical trials. However, the ability of eugenol to induce oxidative stress, as observed in cell-based assays, appears to be related to its cytotoxic and antitumor effect. Other compounds with dual antioxidant and prooxidant effect have a dose/concentration-response relationship, for example, at low doses/concentrations present antioxidant effect and at high doses/concentrations show prooxidant effect [75,76,77,78]; however, we do not find this relationship with the data published with eugenol. Problems related to the degree of purity of the compound, its evaporation (for volatile compounds for example) during the experiments, the methods used to quantify these data (since different cellular and animal models may present divergent results and interpretations) and some laboratory and interpretation errors (including the use of cell lines contaminated with *Mycoplasma* sp., errors in cell line authentication, etc.) may contribute to explain these controversial results. 

In relation to the structure-activity relationship of eugenol, the cytotoxicity of eugenol-related compounds has been associated with the activity of the production of phenoxyl radicals, their stability of the subsequent quinonemethide and the hydrophobicity [79]. In relation to the antioxidant activity, the number of hydroxyl groups in the phenol ring of eugenol enhanced it antioxidant action [31,80]. Moreover, the presence of bromine substituent in ortho-position to the OH-group increases its antioxidant activity [81].

## 4. Conclusions

**Figure 3:**
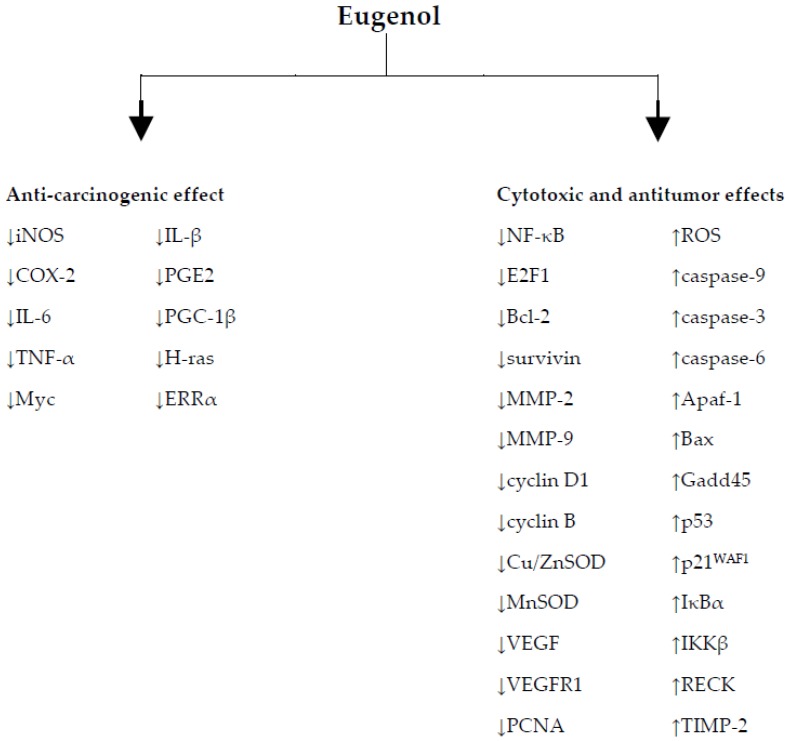
Molecular mechanisms of eugenol. ↑: upregulation; ↓: downregulation; Apaf-1: apoptotic protease activating factor 1; Bax: BCL2 associated X, apoptosis regulator; Bcl-2: B-cell lymphoma 2, apopstosis regulator; COX-2: cycloxygenase-2; Cu/ZnSOD: copper- and zinc-containing superoxide dismutase; ERRα: estrogen-related receptor alpha; Gadd45: growth arrest and DNA damage-inducible 45: IKKβ: IκB kinase α; IL-6: interleukin 6; iNOS: inducible nitric oxide synthase; IκBα: inhibitor of kappa B; MMP-2: matrix metalloproteinase-2; MMP-9: matrix metalloproteinase-9; MnSOD: manganese superoxide dismutase; NF-κB: nuclear factor-kappa B; PCNA: proliferating cell nuclear antigen; PGC-1β: peroxisome proliferator-activated receptor gamma coactivator 1-beta; PGE2: prostaglandin E2; RECK: reversion-inducing-cysteine-rich protein with kazal motifs; ROS: reactive oxygen species; TIMP-2: tissue inhibitor of metalloproteinase-2; TNF-α: tumor necrosis factor alpha; VEGF: vascular endothelial growth factor; VEGFR1: vascular endothelial growth factor receptor 1.

The studies presented in this review reveal the therapeutic potential of eugenol in cancer prevention and treatment and the relationship with its antioxidant and pro-oxidant activities. The Figure 3 summarize the molecular mechanisms of eugenol. Therefore, the consumption of vegetables containing this compound in significant quantities might well be useful in inhibiting the free radicals responsible for tumor development. In addition, the data reported are in accordance with the scientific understanding that a better quality of life and increased longevity may be obtained via healthy food, with the health promoting effects of its bioactive constituents.

## Figures and Tables

**Table 1:** Summary of anti-carcinogenic effect of eugenol.

Carcinogenesis Model	Carcinogen	Eugenol Administration	Effect	References
Skin carcinogenesis	DMBA + TPA	Topical	Reduction in tumor incidence and size; and/or development of papillomatous keratoacanthoma with minimal cell proliferation but without squamous cell carcinoma	[20]
Skin carcinogenesis	DMBA + croton oil	Topical	Inhibition of tumor formation ~60%	[21,22]
Skin carcinogenesis	benzo[a]pyrene	Topical	Inhibition of tumor formation ~50%	[23]
Skin Carcinogenesis	DMBA	Topical	Minimal protection	[24]
Gastric carcinogenesis	MNNG	Intragastric	Inhibition of tumor formation ~75%	[25,26]
Lung carcinogenesis	Urethane	Oral	No protection	[28]

DMBA: 7,12-dimethylbenz[a]anthracene; TPA: 12-otetradecanoylphorbol-13-acetate; MNNG: *N*-methyl-*N*′-nitro-*N*-nitrosoguanidine.

**Table 2:** Summary of in vitro cytotoxic effects of eugenol against cancer and non-cancer cell lines.

Cell Lines	Histological Type	Origin	IC_50_ (µM)	References
*Cancer cells*				
Sbcl2	Primary melanoma	Human	~0.5	[45]
WM3211	Primary melanoma	Human	~0.5	[45]
WM98-1	Primary melanoma	Human	~0.5	[45]
WM1205Lu	Metastatic melanoma	Human	~0.5	[45]
SK-Mel-28	Melanoma	Human	7.2	[51]
A2058	Melanoma	Human	12.2	[51]
WM266-4	Melanoma	Human	>100	[64]
SK-Mel-28	Melanoma	Human	>100	[64]
LCP-Mel	Melanoma	Human	>100	[64]
LCM-Mel	Melanoma	Human	>100	[64]
PNP-Mel	Melanoma	Human	>100	[64]
CN-MelA	Melanoma	Human	>100	[64]
13443	Melanoma	Human	>100	[64]
GR-Mel	Melanoma	Human	>100	[64]
HSG	Submandibular gland adenocarcinoma	Human	~100	[59]
			396	[60]
T47-D	Breast carcinoma	Human	0.9	[50]
MDA-MB-231	Breast adenocarcinoma	Human	1.7	[50]
			15.1	[51]
			~1600	[71]
MCF-7	Breast adenocarcinoma	Human	1.5	[50]
			22.8	[51]
			~400	[70]
			900	[71]
HCT-15	Colon adenocarcinoma	Human	300	[69]
HT-29	Colon adenocarcinoma	Human	500	[69]
Caco-2	Colon carcinoma	Human	~750	[73]
SNU-C5	Colon carcinoma	Human	129.4	[46]
LNCaP	Prostate adenocarcinoma	Human	~550	[49]
PC-3	Prostate carcinoma	Human	~180	[49]
DU-145	Prostate carcinoma	Human	30.4	[48]
SIHA	Cervical carcinoma	Human	18.3	[51]
HeLa	Cervical carcinoma	Human	500	[72]
HepG2	Hepatocellular carcinoma	Human	118.6	[46]
			~500	[73]
3LL	Lewis lung carcinoma	Mouse	89.6	[46]
KB	Oral squamous cell carcinoma	Human	28.5	[48]
HSC-2	Oral squamous cell carcinoma	Human	~700	[72]
HOS	Osteosarcoma	Human	1500	[66]
HL-60	Promyelocytic leukemia	Human	23.7	[46]
			380	[61]
U-937	Histocytic lymphoma	Human	39.4	[46]
*Non-cancer cells*				
MCF 10A	Breast epithelial	Human	2.2	[50]

IC_50_: half maximal inhibitory concentration.

**Table 3:** Summary of in vivo antitumor effect of eugenol.

Tumor	Histological Type	Origin	Dose (mg/kg)	Treatment	Route	Inhibition Rate (%)	References
B16	Melanoma	Mouse	125	Twice a week	i.p.	62	[45]
Ehrlich (ascites model)	Carcinoma	Mouse	100	Every two days for four weeks	i.p.	28.9	[74]
Ehrlich (solid model)	Carcinoma	Mouse	100	Every two days for four weeks	i.p.	24.4	[74]
MDA-MB-231	Breast adenocarcinoma	Human	100	Every two days for four weeks	i.p.	~66	[50]

i.p.: intraperitoneal.

## Abbreviations

Apaf-1	Apoptotic protease activating factor 1
Bax	BCL2 associated X, apoptosis regulator
Bcl-2	B-cell lymphoma 2, apopstosis regulator
COX-2	Cyclooxygenase-2
CYP 1A1	Cytochrome P450 family 1 subfamily A member 1
CYP1B1	Cytochrome P450 family 1 subfamily B member 1
DFF45	DNA fragmentation factor 45
DMBA	7,12-dimethylbenz[a]anthracene
DNA	Deoxyribonucleic acid
DPPH	2,2-diphenyl-1-picrylhydrazyl
ER	Endoplasmic reticulum
Gadd45	Growth arrest and DNA damage-inducible 45
IC_50_	Half maximal inhibitory concentration
IKKβ	Inhibitor of kappa B
IL-6	Interleukin-6
iNOS	Inducible nitric oxide synthase
IκBα	IκB Kinase α
MA	Malonaldehyde
MCF 10A-ras	H-ras transfected MCF 10A
MMP	Matrix metalloproteinases
MNNG	*N*-Methyl-*N*′-nitro-*N*-nitrosoguanidine
NF-κB	Nuclear factor kappa B
PARP	Poly(ADP-ribose)polymerase
PCNA	Proliferating cell nuclear antigen
PGE2	Prostaglandin E2
RECK	Reversion-inducing-cysteine-rich protein with kazal motifs
ROS	Reactive Oxygen Species
SGOT	Serum glutamic-oxaloacetic transaminase
TA	Thioacetamide
TBARS	Thiobarbituric acid-reactive substances
TIMP-2	Tissue inhibitor of metalloproteinase-2
TNF-α	Tumor necrosis factor alpha
TPA	12-otetradecanoylphorbol-13-acetate
VEGF	Vascular endothelial growth factor
VEGFR1	Vascular endothelial growth factor receptor 1
